# Engineering siRNA therapeutics: challenges and strategies

**DOI:** 10.1186/s12951-023-02147-z

**Published:** 2023-10-18

**Authors:** Syed Saqib Ali Zaidi, Faria Fatima, Syed Aqib Ali Zaidi, Dezhong Zhou, Wuquan Deng, Shuai Liu

**Affiliations:** 1 School of Chemical Engineering and Technology, Xi’an Jiaotong University, Xi’an, 710049 China; 2 College of Medical Technology, Ziauddin University, Karachi, 74700 Pakistan; 3 Shenzhen Key Laboratory of Anti-Aging and Regenerative Medicine, Shenzhen University Medical School, Shenzhen University, Shenzhen, 518060 China; 4 Department of Endocrinology and Metabolism, Chongqing Diabetic Foot Medical Research Center, Chongqing University Central Hospital, Chongqing Emergency Medical Center, Chongqing, 400014 China; 5 College of Pharmaceutical Sciences, Zhejiang University, Hangzhou, 310058 China

**Keywords:** siRNA, Extracellular barriers, Intracellular barriers, siRNA modification, siRNA delivery

## Abstract

Small interfering RNA (siRNA) is a potential method of gene silencing to target specific genes. Although the U.S. Food and Drug Administration (FDA) has approved multiple siRNA-based therapeutics, many biological barriers limit their use for treating diseases. Such limitations include challenges concerning systemic or local administration, short half-life, rapid clearance rates, nonspecific binding, cell membrane penetration inability, ineffective endosomal escape, pH sensitivity, endonuclease degradation, immunological responses, and intracellular trafficking. To overcome these barriers, various strategies have been developed to stabilize siRNA, ensuring their delivery to the target site. Chemical modifications implemented with nucleotides or the phosphate backbone can reduce off-target binding and immune stimulation. Encapsulation or formulation can protect siRNA from endonuclease degradation and enhance cellular uptake while promoting endosomal escape. Additionally, various techniques such as viral vectors, aptamers, cell-penetrating peptides, liposomes, and polymers have been developed for delivering siRNA, greatly improving their bioavailability and therapeutic potential.

## Introduction

**Fig. 1:**
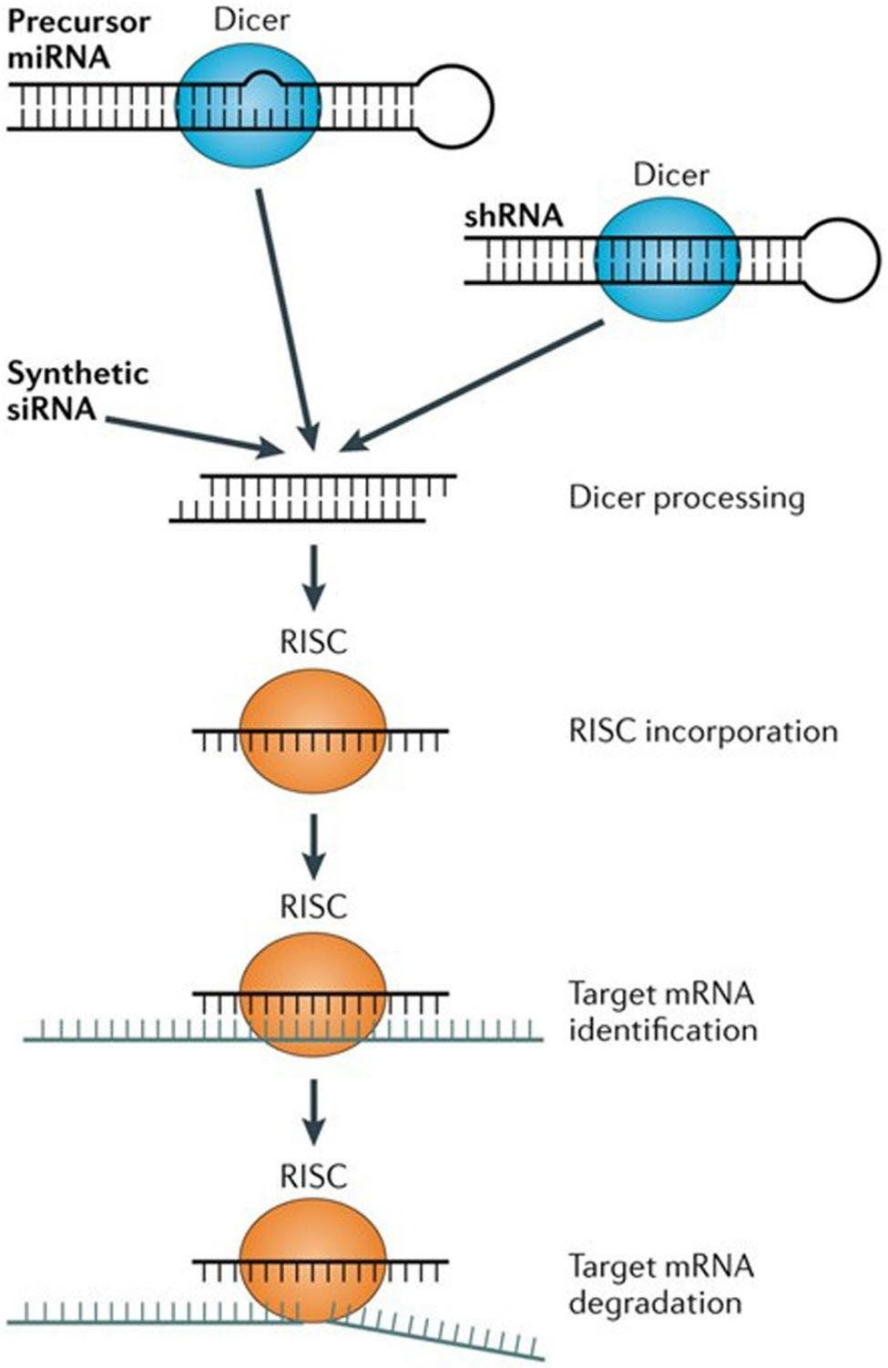
RNAi biogenesis and mechanism. [12]

RNA interference (RNAi) has become the primary method for selective and targeted gene silencing while preserving intracellular gene expression mechanisms [1]. Ambros et al. first explored RNAi by discovering that the binding of RNA transcripts to the lin-4 gene could reduce lin-14 protein expression in Caenorhabditis elegans [2, 3]. This discovery led to similar RNAi pathways in other organisms such as fungi, plants, insects, animals, and humans [4]. RNAi primarily involves small double-stranded noncoding RNA called siRNA and miRNA [5]. The siRNA are commonly used in mammalian cells and are triggered by the introduction of a chemically synthesized long double-stranded RNA into the cytoplasm, which is then cleaved into siRNA of about 19–23 nucleotides (nt) by the RNase III enzyme Dicer [4, 6, 7]. The guide strand of mature siRNA is loaded into the siRNA-induced silencing complex (siRISC), where it combines with Argonaute (AGO) family proteins that help locate the 3’ untranslated regions (UTRs) of mRNA having siRNA complementarity [8]. Thus, the siRISC binding with the complementary mRNA prevents translational expression and promotes target mRNA degradation (Fig. 1) [6, 8]. However, miRNA are endogenous small noncoding transcripts of 18–22 nt that post-transcriptionally obstruct the regulation of gene expression *via* sequence-specific means [9]. miRNAi is initiated by their expression from the genome as dsRNA following enzymatic action in the nucleus to produce premature microRNA (pre-miRNA) of about 70 nt [10]. Despite small differences in initial processing routes, both siRNA and miRNA follow comparable downstream pathways [5]. In addition, miRNA depend on partial complementarity for mRNA binding, while siRNA rely on 100% complementarity [6]. It is worth noting that a single siRNA or miRNA can bind to several mRNA targets, but their intracellular enzymatic activity eventually destroys them after dilution beyond the therapeutic level [11].

It has previously been documented that siRNA’ capacity to probe gene functions allows them to suppress the expression of target genes in a variety of incurable diseases, such as Mendelian disorders [13], liver cirrhosis [14], viral infections [15], hypercholesterolemia [16], and cancers [17]. The first US FDA-approved siRNA-based RNAi therapeutic (i.e., Patisiran, Alnylam Pharmaceuticals [18]) marked a significant breakthrough in August 2018, ushering in a new era of drug development [19]. The Patisiran (ONPATTRO®) was proposed for the medication of hereditary transthyretin-mediated amyloidosis by targeting a sequence of transthyretin-mRNA [20]. Later in November 2019, Alnylam Pharmaceuticals launched Givosiran (Givlaari™), the second US FDA-approved siRNA-based therapeutic for adults dealing with acute hepatic porphyria (AHP). The Givosarin (Givlaari™) induction into medicinal treatment bring a new hope of confidence in siRNA based therapeutics and thus multiple siRNA based drugs are in different clinical phases [18].

Although, number of advancements and a huge investment have been made to bring siRNAs in commercial market, the intrinsic properties of siRNA constrains its availability and application in clinical therapy [7]. Here, we aim to thoroughly elucidate these obstacles and constraints that siRNA therapeutics confront prior to and after cellular uptake. Furthermore, recent approaches and improved pathways to overcome these obstacles are also discussed.

## Physiochemical and biological challenges for the delivery of siRNA-based therapeutics

While siRNA are highly effective at post-transcriptionally suppressing the expression of desired target genes, and their *in-vivo* delivery faces significant obstacles such as off-target interactions, determining the optimal administration route, limited circulation half-life, inadequate endosomal escape into the cytosol, renal clearance, and immune evasion [21]. Furthermore, siRNA’ intrinsic characteristics, specifically their potent anionic charge and exceptional hydrophilicity, also render them susceptible to systematic degradation within biological systems [22].

### Extracellular challenges

Therapeutic siRNA can be administered either locally or systemically, or a combination of both in some clinical trials. Local administration provides direct access of therapeutic siRNA to the specific tissue and organ, bypassing challenges posed by systemic delivery. The primary methods for local administration are intranasal, inhalation, and intra-tracheal pathways. However, despite being less complex than systematic delivery, the local approach has several additional obstacles that need to be overcome [23].

In the systematic administration of siRNA, unmodified and unprotected siRNA may undergo degradation upon intravenous delivery into the circulatory system, primarily due to the presence of endonucleases in the serum. The resulting degradation byproducts may trigger an immune response, leading to undesirable immunological reactions [24]. Additionally, the immune system can identify siRNA as foreign RNA, prompting the production of cytokines and interferons that cause off-target effects and toxicity. Several variables affect the immunogenic potential of siRNA, such as its length and sequence, the delivery method used, and the patient’s immunological profile [25]. Pattern recognition receptors (PRRs) like Toll-like receptors (TLRs) and retinoic acid-inducible gene I (RIG-I)-like receptors (RLRs) play a crucial role in recognizing siRNA as foreign RNA, initiating the production of inflammatory cytokines and type I interferons (IFNs) [26].

As previously mentioned, synthetic siRNAs are short in length (19–23 nt) and have a low molecular weight. This results in swift elimination through the renal system and a short half-life in the bloodstream, ranging from 6 min to 1 h [27, 28]. In addition, the highly anionic nature of siRNAs encourages activation of the reticuloendothelial system (RES), also known as the mononuclear phagocytic system, leading to opsonization-mediated clearance of siRNAs from circulation, especially when coupled with synthetic delivery vehicles [29]. Another way that siRNAs are cleared is through receptor-mediated uptake by RES cells expressing specific receptors such as scavenger receptors and mannose receptors [30]. To improve the pharmacokinetic properties of siRNAs and minimize their clearance by the RES, various modifications have been made to the siRNA structure. One approach is to introduce polymers, like PEG, to decrease the recognition of siRNAs by serum proteins and RES receptors [31]. Another method is to use delivery systems like lipid-based nanoparticles to shield siRNAs from RES receptors and increase their circulation time [32]. Chemical conjugates, like cholesterol, have also successfully been used to enhance siRNA stability and reduce clearance. These modifications have effectively increased siRNA pharmacokinetics and decreased clearance by the RES across multiple studies [32, 33].

Non-target cells, including immune cells and non-cancerous cells, can take up siRNA through various methods such as endocytosis and phagocytosis. This uptake can inadvertently result in gene silencing and toxicity, leading to undesirable side effects [34]. To address these non-specific interactions, several techniques have been developed. These include chemical modifications of siRNA to improve its stability and reduce protein binding, packaging siRNA into nanoparticles to promote delivery and inhibit non-target cell uptake, and using tissue-specific targeting ligands to optimize siRNA specificity and prevent off-target effects [35].

### Intracellular challenges

**Fig. 2:**
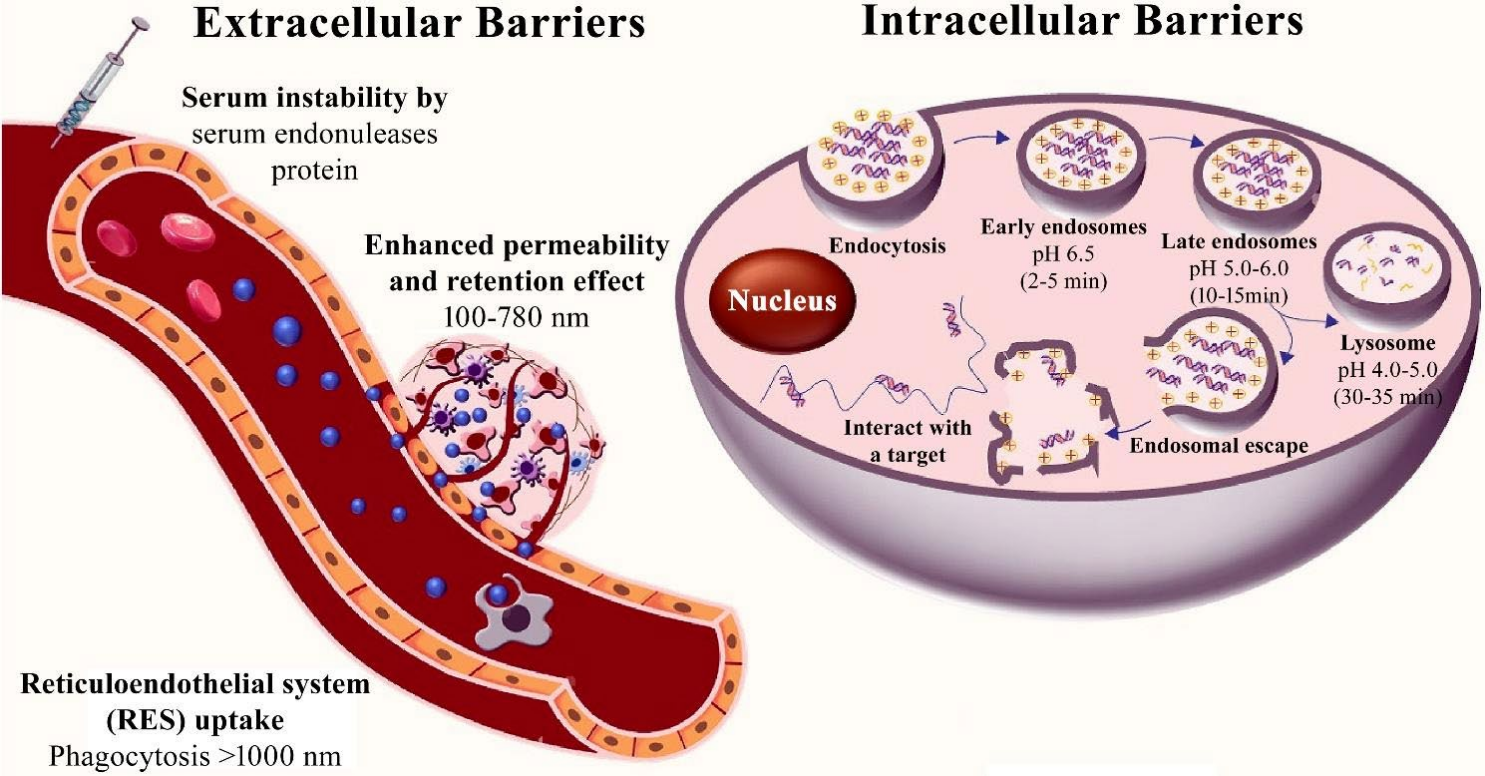
Extracellular and intracellular barriers for siRNA therapeutics. [23]

The delivery of siRNA into target cells is a complex process that involves overcoming several intracellular barriers, including cell membrane permeability, endosomal escape, degradation by nucleases, and intracellular trafficking (Fig. 2). Overcoming these barriers is crucial for developing effective siRNA therapeutics.

However, delivering siRNA therapeutics to target cells is a significant challenge due to the hydrophilic and negatively charged nature of siRNA molecules. This makes it difficult for them to passively diffuse through the hydrophobic lipid bilayer of the plasma membrane [36]. In addition, the size, charge, and hydrophilicity of siRNA molecules create obstacles to their cellular uptake by repelling negatively charged phospholipids in the plasma membrane and being larger than most membrane channels and transporters [37].

Once inside cells, siRNA molecules are enclosed in endosomes, which are membrane-bound vesicles responsible for sorting and transporting cellular material [38]. The endosomal maturation process involves several stages, including early and late endosomes, and lysosomes. During maturation, the pH within the endosomal lumen decreases, leading to the release of siRNA from its delivery vehicle into the cytoplasm. However, siRNA efficacy can be limited by degradation caused by endosomal nucleases and sequestration in non-productive compartments [39, 40]. Larger siRNA molecules are more likely to become entrapped, while cationic materials used for delivery can bind to the endosomal membrane, resulting in entrapment [41]. In addition, serum in the extracellular environment can also contribute to siRNA entrapment, leading to the formation of protein-siRNA complexes [42].

There are two distinct mechanisms that enable siRNA to escape endosomes: membrane fusion and membrane disruption. During membrane fusion, endosomes containing siRNA fuse with the plasma membrane, releasing the siRNA into the cytoplasm. This process involves the fusion of endosomal membranes with lysosomal membranes or the plasma membrane, requiring the participation of proteins such as Rab GTPases, SNAREs, and ESCRT [43, 44]. In contrast, the membrane disruption mechanism destabilizes siRNA-loaded endosomes by forming pores in the endosomal membrane. This is mediated by cationic peptides or polymers that interact with the negatively charged endosomal membrane, leading to its disruption and release of siRNA into the cytoplasm [41].

Several strategies have been developed to address the challenge of endosomal escape, including pH-sensitive liposomes, cell-penetrating peptides, endosomal disruptors, and nanoparticles [45]. The pH-sensitive liposomes are engineered to respond to changes in pH inside the endosome, making them unstable and causing them to rupture, thereby releasing siRNA into the cytoplasm [46]. Cell-penetrating peptides and endosomal disruptors assist in destabilizing endosomal membranes, allowing siRNA to pass through the cell membrane and enter the cytosol. Nanoparticles are also a promising approach that promotes endosomal escape through multiple mechanisms, such as the proton sponge effect, osmotic swelling, and membrane fusion. The proton sponge effect involves nanoparticles being taken up into the endosome, causing an influx of protons, leading to endosomal swelling and rupture [47]. Osmotic swelling occurs when nanoparticles induce osmotic pressure, resulting in endosomal swelling and rupture [48]. Finally, membrane fusion utilizes nanoparticles that combine with endosomal membranes, ultimately resulting in siRNA release into the cytoplasm, where it integrates with RNAi machinery [49].

After endosomal escape, siRNA interacts with a variety of intracellular components such as RISC, cytoplasmic RNA-binding proteins, and other RNA-binding proteins. The fate of siRNA depends on its interactions with these components and can be broadly categorized into two pathways: the productive pathway and the non-productive pathway [50]. In the productive pathway, siRNA binds to RISC, which incorporates it into the RISC-loading complex (RLC). The RLC unwinds the siRNA duplex, selects the guide strand, and loads it onto the mature RISC complex. The guide strand directs RISC to its complementary mRNA target, where RISC cleaves the mRNA, leading to gene silencing. The cleaved mRNA is subsequently degraded, decreasing the expression of the target protein. The RISC complex repeatedly cleaves mRNA, thus sustaining gene silencing [51]. In contrast, in the non-productive pathway, siRNA may be sequestered into non-functional compartments, such as stress granules, processing bodies, or cytoplasmic bodies. These compartments can form under various stress conditions and may trap siRNA molecules, preventing them from participating in productive pathways [52]. Moreover, cytoplasmic exonucleases can degrade siRNA, reducing its concentration and activity [53].

## Strategies for engineering siRNA therapeutics

Engineering siRNA for enhanced delivery is crucial, and several modifications have been developed to improve siRNA stability, cellular uptake, and intracellular distribution. These modifications can be broadly categorized into two classes: chemical modifications and formulation-based modifications. Chemical modifications involve altering the molecular structure of siRNA, while formulation-based modifications use a delivery vehicle to encapsulate siRNA or modify its formulation to enhance delivery efficacy [54].

### Chemical modification

Chemical modifications have been utilized to improve the stability, reduce the immunogenicity, and enhance cellular uptake of siRNA molecules. These modifications can be categorized into two groups: nucleotide modifications and phosphate backbone modifications [55].

#### Nucleotide modifications

**Fig. 3:**
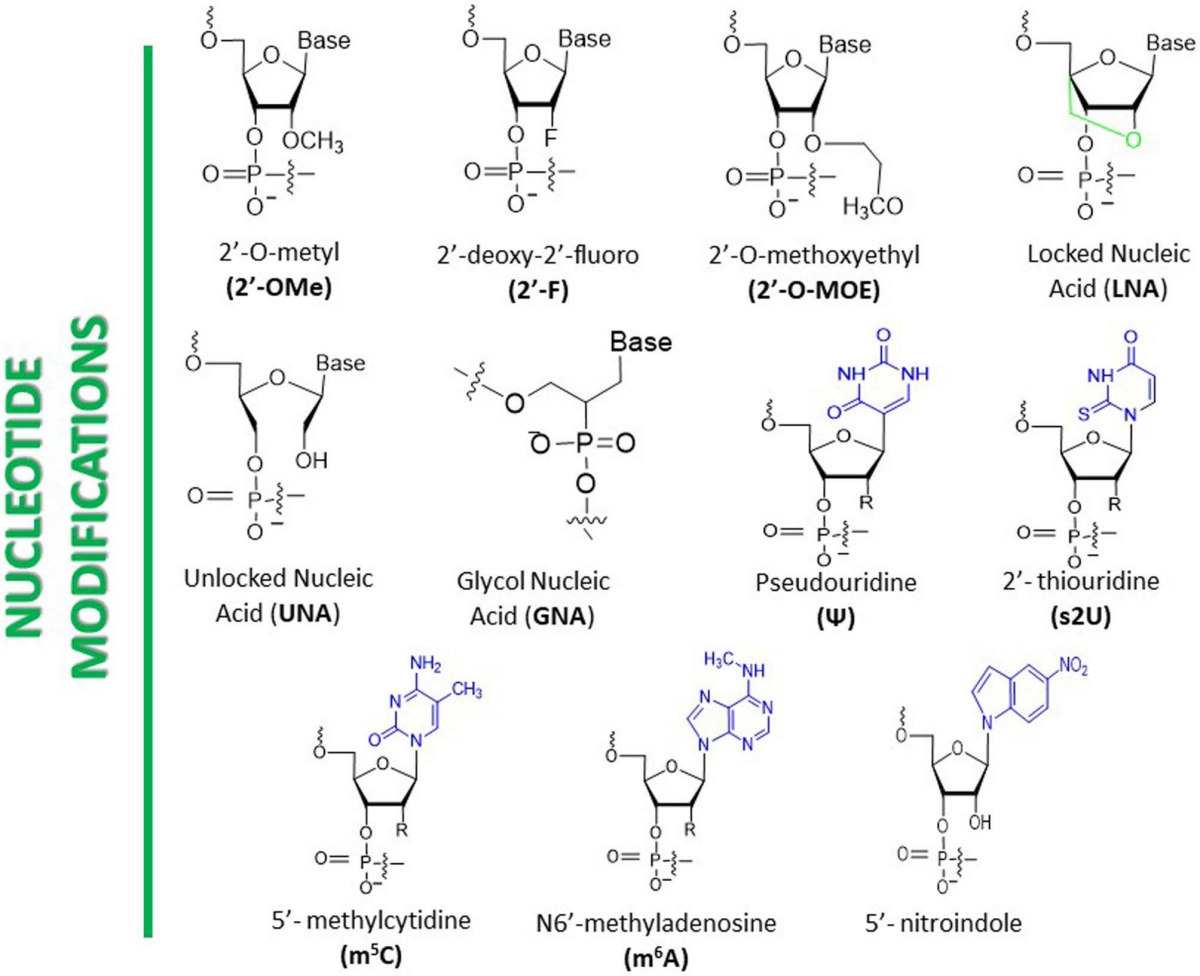
Nucleotide modifications of siRNA. [6, 56]

Chemical modifications can be performed on nucleotide bases, sugar moiety, or nucleosides based on the inherent structure of ribonucleotides. Substituting uridine with 2’-O-methyl uridine (2’-OMe U) is a commonly used base modification in siRNA, which has been shown to increase siRNA stability and reduce off-target effects (Fig. 3) [56]. Another modification that replaces cytidine with 2’-deoxy-2’-fluoro cytidine (2’-F C) has also been found to improve siRNA stability and minimize off-target effects [57]. Additional base modifications include replacing adenosine with N6-Methyladenosine (m6 A) [58], guanosine with 2’-O-methyl guanosine (2’-OMe G) [5], and uridine with 2-thiouridine (2-S U) [59]. Sugar modifications frequently employed in siRNA involve substituting ribose with 2’-O-methyl ribose (2’-OMe R) or 2’-fluoro ribose (2’-F R), which have exhibited better siRNA stability and decreased immunogenicity [60]. Other sugar modifications include replacing ribose with locked nucleic acid (LNA), unlocked nucleic acid (UNA), glycol nucleic acid (GNA), or 2’-O-methoxyethyl (2’-MOE) sugar [60–62]. The most commonly utilized nucleoside modification in siRNA is the incorporation of a 5’-triphosphate cap, which has been shown to enhance siRNA activity by facilitating its loading onto the RNA-induced silencing complex (RISC) [63]. Another nucleoside modification involves attaching a cholesterol moiety to siRNA, improving its cellular uptake, and promoting its accumulation in target tissues [64].

#### Phosphate-backbone modification

**Fig. 4:**
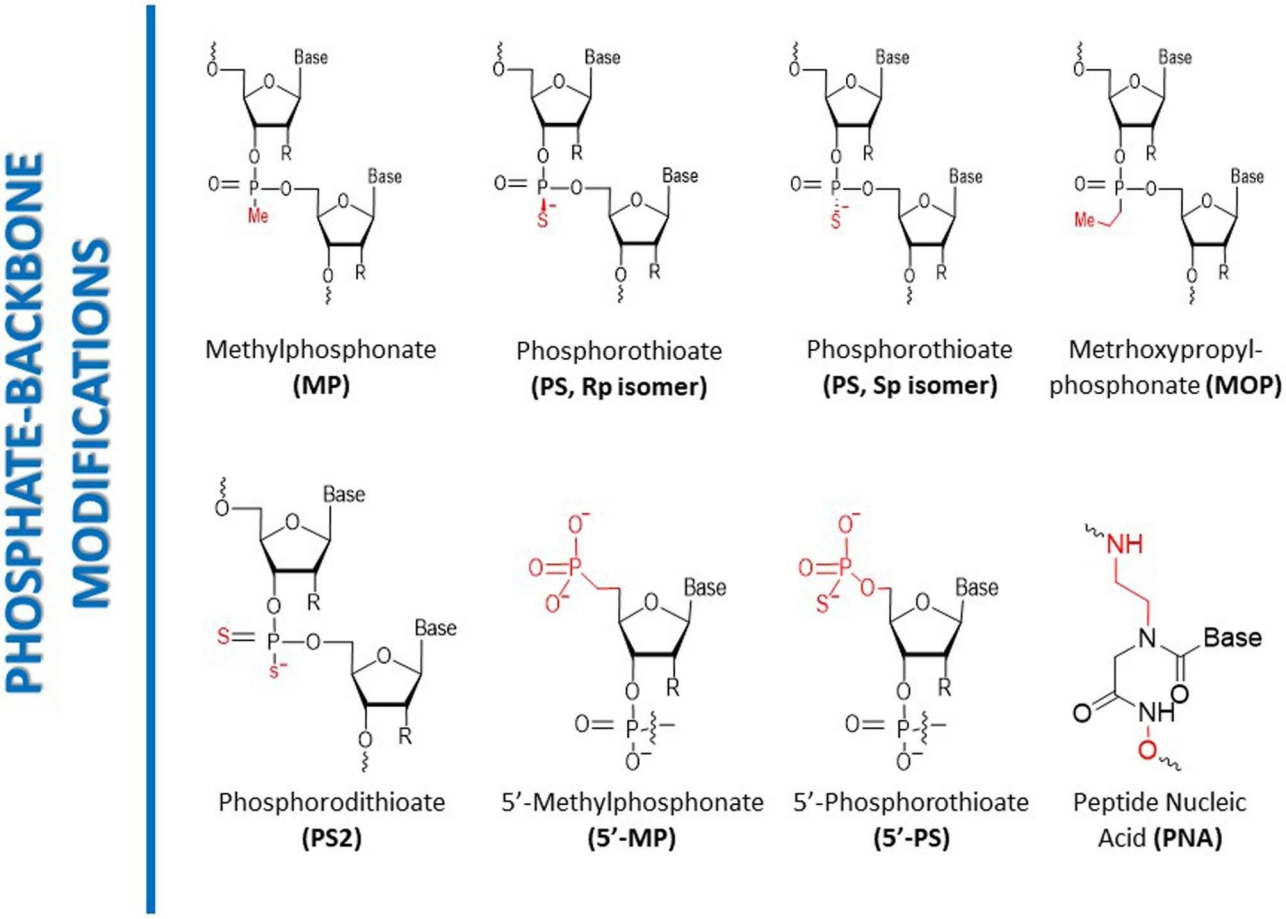
Phosphate-backbone modifications of siRNA. [6, 56]

The structural foundation of siRNA typically consists of phosphodiester bonds, which are vulnerable to breakdown by nucleases [65]. To address this issue, phosphonate backbone modifications can be employed by substituting the phosphodiester bonds with phosphonate linkages that are resistant to nuclease degradation (Fig. 4). One common type of phosphonate backbone modification is the addition of a methylphosphonate (MeP) linkage, which replaces one of the non-bridging oxygen atoms of the phosphodiester bond with a methyl group, creating a non-ionic and chemically stable linkage [66]. Another modification, phosphorodithioate (PS2) linkage, replaces both non-bridging oxygen atoms of the phosphodiester bond with sulfur atoms, producing a chemically stable and nuclease-resistant linkage. PS2-modified siRNA has demonstrated improved stability and efficacy compared to unmodified siRNA and has been effective in preclinical models of cancer and viral infections [67]. Phosphorothioate (PS) backbone modification is another commonly used phosphonate modification. It substitutes the non-bridging oxygen atom of the phosphodiester bond with a sulfur atom, creating a negatively charged phosphorothioate linkage. While PS-modified siRNA is more stable and resistant to nuclease degradation than unmodified siRNA, it can cause off-target effects and immune stimulation, limiting its therapeutic potential [68]. Other types of phosphonate modifications have also been researched, including phosphoramidate (PA) and phosphoroselenoate (PSe) linkages. The phosphonate backbone of siRNA can also be combined with other modifications, such as sugar modifications, to enhance the stability, efficacy, and specificity of siRNA. Integrating MeP or PS2 backbone modifications with 2’-O-methyl or 2’-fluoro sugar modifications can confer better pharmacokinetic and pharmacodynamic characteristics upon siRNA, leading to improved therapeutic outcomes. Additionally, these modifications can reduce off-target effects and immune stimulation, improving the therapeutic potential of siRNA [69, 70].

### Formulation-based delivery of siRNA therapeutics

To enhance the efficacy of siRNA, formulation-based modifications can be used to protect it from degradation and improve cellular uptake. Lipid-based formulations, such as liposomes and LNP, are commonly utilized due to their biocompatibility, ease of formulation, and tissue-targeting ability. These formulations can encapsulate siRNA, shield it from enzymatic degradation, and improve its cellular uptake through receptor-mediated endocytosis. Polymer-based nanoparticles offer tunable size and surface charge for efficient cellular uptake and targeting, while viral vectors such as adeno-associated virus (AAV) and lentivirus have high transduction efficiency and the ability to integrate siRNA into the host genome [71–73].

#### Viral delivery systems

The delivery of siRNA into target cells remains a significant challenge in RNAi technology. Viral vectors have been developed as effective systems for delivering siRNA due to their ability to infect a wide range of cell types and high transduction efficiency. Various types of viral delivery systems have been developed for siRNA, including adenoviruses, retroviruses, lentiviruses, AAVs, and herpes simplex viruses (HSVs), each with unique features and mechanisms suitable for siRNA delivery to specific target cells [74]. The entry of viral vectors into the target cells depends on the type of vector. For instance, adenoviral vectors enter via receptor-mediated endocytosis while retroviral and lentiviral vectors enter via receptor-mediated binding and membrane fusion [24]. However, the endosomal pathway poses a significant barrier for successful siRNA delivery by viral vectors. To overcome these obstacles, viral vectors have developed various mechanisms for endosomal escape, including pH-dependent disruption, fusion with the endosomal membrane, and interaction between the viral capsid and the endosomal membrane. Once in the cytoplasm, the viral vector must release the siRNA to begin RNAi, which depends on the design of the siRNA expression cassette [75–77].

##### Adenoviral vector

Adenoviruses are double-stranded DNA viruses that can infect both humans and animals, and they are commonly used in gene therapy and vaccination. Adenoviral vectors have been demonstrated to be effective carriers for siRNA delivery since they can transduce a broad range of cell types and carry large transgene cassettes. Adenoviral vectors enter cells via cell surface receptors, and once inside the cell, the viral DNA is transported to the nucleus where it is transcribed and replicated. However, one significant challenge in delivering siRNA by adenoviral vectors is efficiently releasing the siRNA from the endosome to the cytoplasm [78]. To overcome this obstacle, adenoviral vectors have developed various strategies for endosomal evasion, including a pH-sensitive approach mediated by viral protein VI and induction of auto phagosomes [79, 80]. Nevertheless, using adenoviral vectors for siRNA delivery may cause toxicity and induce immune responses, which affects its efficacy [81].

##### Retroviral vector

Retroviral vectors are commonly used in gene therapy to provide long-term gene expression by integrating into the host genome. They are enveloped viruses with a single-stranded RNA genome that undergo reverse transcription to form double-stranded DNA, which then integrates into the host genome. Recently, retroviral vectors have been studied as potential vehicles for delivering siRNA to downregulate specific gene expression. Retroviral vectors offer advantages such as high transduction efficiency and long-term gene expression [82, 83]. However, using retroviral vectors for siRNA delivery may result in off-target effects due to their integration into the host genome. To improve the safety profile of retroviral vectors for siRNA delivery, two approaches have been developed. The first approach involves designing self-inactivating (SIN) vectors that contain a deletion in the U3 region of the long terminal repeat (LTR). This deletion inactivates the promoter and enhancer elements in the 3’ LTR, reducing the risk of insertional mutagenesis [84, 85]. The second approach involves developing lentiviral vectors, a subclass of retroviral vectors that can transduce non-dividing cells and have a broader host range. Lentiviral vectors can be pseudo-typed with different envelope proteins to target specific cell types, increasing their specificity and reducing off-target effects. In addition, lentiviral vectors have a lower risk of insertional mutagenesis because of their integration preference for active genes. These modifications have improved the safety and specificity of retroviral vectors for siRNA delivery, making them a promising approach for gene therapy applications [86, 87].

##### Adeno-associated viral (AAVs) vector

AAV is a promising viral vector for gene therapy and RNAi applications due to its low immunogenicity, ability to transduce both dividing and non-dividing cells, and long-term gene expression [88]. AAV-mediated siRNA delivery involves using recombinant AAV vectors that carry a therapeutic siRNA cassette under the control of a tissue-specific promoter to silence target genes. The AAV vector is produced by co-transfecting a packaging cell line with the AAV vector plasmid and a helper plasmid that provides the missing viral genes in trans., followed by purification from the cell lysate [89, 90]. Despite its advantages, AAV-mediated siRNA delivery encounters hurdles such as limited packaging capacity, off-target effects, and lack of efficacy in specific cell types [91]. To address these obstacles, various approaches have been developed, including introducing mutations into AAV vectors to enhance transduction efficiency and reduce immunogenicity and utilizing self-complementary AAV (scAAV) for siRNAs to increase potency [92]. However, despite these challenges, the benefits of AAV-mediated siRNA delivery outweigh the drawbacks. The low immunogenicity of AAV allows for repeated dosing, while its ability to transduce both dividing and non-dividing cells makes it a suitable vector for siRNA delivery to post-mitotic tissues such as neurons and muscle cells. Additionally, AAV can achieve long-term gene expression in the target tissue, making it a promising tool for gene therapy and RNAi applications [89, 93, 94].

AAV has potential applications in siRNA therapy for a range of diseases, including viral infections, genetic disorders, cancer, and neurodegenerative diseases. By modifying AAVs to express siRNA molecules that target specific genes, AAV-delivered siRNAs can prevent viral replication, reduce the production of disease-causing proteins, inhibit tumor growth, and slow or reverse disease progression. These approaches have been explored in studies and have shown promising results in preclinical and clinical settings, demonstrating the potential of AAV-based siRNA therapies for treating various diseases [93, 95, 96].

##### Herpes simplex viruses (HSVs) vector

HSVs are a cluster of viral particles that can cause various infections, ranging from cold sores to genital herpes. HSVs have the ability to establish a latent infection in the host, which can later trigger recurrent outbreaks. This characteristic has piqued the interest of researchers who are investigating their potential as vectors for delivering therapeutic agents, such as siRNAs. HSVs are suitable candidates for siRNA delivery due to their capacity to infect a wide range of cell types and establish persistent latent infections [97, 98]. In addition, HSVs have a large genome that can accommodate foreign DNA sequences, making them an optimal platform for gene delivery. By engineering HSVs to express siRNAs that selectively target viral genes, viral replication and transmission can be inhibited. HSVs can also be designed to target specific cell types, enabling targeted delivery of siRNAs to infected cells [97, 99].

Numerous studies have explored the use of HSVs as siRNA carriers for treating viral infections, including those caused by HSV-1 and HSV-2 [100]. HSVs naturally target cancer cells and can invade and reproduce selectively in tumor cells while preserving normal cells. Furthermore, HSVs can be genetically modified to express therapeutic genes or siRNAs that specifically target cancer cells, causing tumor cell death. Multiple studies have proven the potential of HSVs as siRNA carriers for treating various cancers, such as breast cancer, melanoma, and glioblastoma [101, 102].

However, HSV-based siRNA delivery systems have limitations and challenges. One significant concern is the possibility of the virus reactivating and causing disease in the host, despite establishing long-term latent infections. This can result in repeated herpes outbreaks [103]. In addition, the host immune response may impair the efficacy of the virus as a gene therapy vector. The immune system can identify and clear the virus, restricting its ability to transport siRNAs to target cells [102].

#### Non-viral delivery systems

Although viral vectors have been shown to effectively deliver siRNA and have the potential to cure numerous diseases, there is a need for alternative delivery systems that can overcome current limitations and improve efficacy. Non-viral delivery systems for siRNA have emerged as a promising alternative by offering a safer and more efficient approach to delivering siRNA to target cells. These systems utilize various nanoparticle-based strategies, including lipid-based, cell-penetrating peptides, polymeric, and inorganic delivery systems [104]. Lipid-based delivery systems, such as liposomes and solid lipid nanoparticles, can encapsulate siRNA in a protective lipid bilayer, enhancing their stability and cellular internalization [105]. Polymeric delivery systems, such as polyethyleneimine and chitosan, can be designed to deliver siRNA specifically to certain cell types [106]. Inorganic delivery systems, such as gold and silica nanoparticles, can be engineered to functionalize siRNA with targeting ligands for precise delivery [72]. Non-viral delivery systems have exhibited significant potential in delivering siRNA to a wide range of cell types in vitro and in vivo, including cancer and immune cells. However, the efficacy of these systems can be further enhanced by addressing issues related to toxicity and immune response [22].

##### Lipid-based delivery systems

**Fig. 5:**
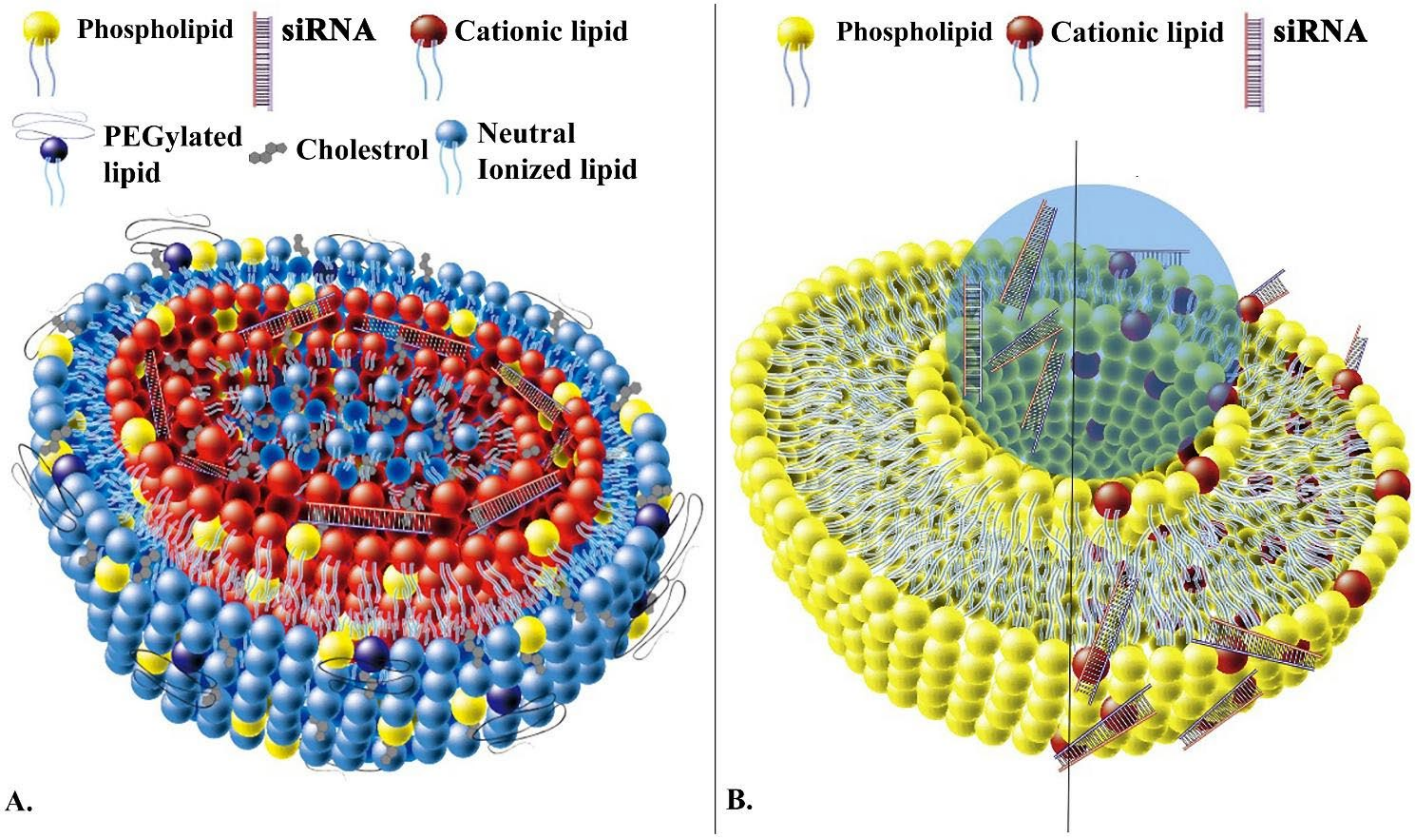
LNPs for siRNA delivery. **(A)** siRNA-encapsulated in core of LNPs. **(B)** liposome-mediated delivery of siRNA. Liposomes’ interior water phase encapsulates siRNA or siRNA binds to cationic lipid-containing liposomes. [105]

Lipid-based delivery systems offer an effective approach for delivering siRNA by encapsulating it within a protective lipid bilayer, which enhances its stability and protects it from degradation (Table 1; Fig. 5). Lipid-based nanoparticles, such as liposomes and solid lipid nanoparticles, are commonly used as carriers for siRNA delivery due to their biocompatibility, low toxicity, and capacity to transport both hydrophilic and hydrophobic siRNA molecules [105]. The lipid bilayer can also be functionalized with ligands to improve targeting and uptake of specific cells or tissues. Lipid-based delivery systems have advantages over other delivery systems, such as viral vectors, including lower immunogenicity, larger cargo capacity, and easier synthesis and modification [107].

**Table 1:** LNPs for the delivery of siRNA

LNPs	Shape	Type of study	Aid	Ref.
DOPE, DOTMA, DPPE-PEG 2000	Spherical	*In-vivo*	Receptor-mediated targeted delivery	[108]
Toc-siRNA	Spherical	*In-vivo*	Improved stability and cleaving efficiency of siRNA, targeted delivery, no side effects	[109]
DLinDAP, DLinDMA, DLinKDMA, or DLinKC2-DMA	Spherical	*In-vitro*		[110]
AtuFECT01	Spherical	*In-vivo* and *In-vitro*	Targeted delivery, stable in bloodstream	[111]

Recent advances in lipid-based delivery systems for siRNA aim to enhance the specificity, stability, and efficiency of siRNA delivery to target cells. Hybrid lipid-based nanoparticles that combine different lipid formulations have been created, such as a cationic and fusogenic lipid hybrid nanoparticle that showed superior gene silencing efficacy and reduced toxicity in cancer cells. Stimuli-responsive lipids have been used to develop pH-sensitive lipid nanoparticles that selectively release siRNA in the acidic environment of tumor cells, enhancing gene silencing [112]. Targeting ligands have also been incorporated into lipid-based nanoparticles to improve the specificity of siRNA delivery, leading to enhanced gene silencing and inhibition of tumor growth [113]. Furthermore, lyophilization has been used to improve the stability of lipid-based nanoparticles during storage and circulation in the bloodstream. For instance, a lyophilized lipid nanoparticle for delivering siRNA targeting cancer cells exhibited high stability and preserved gene silencing efficacy after reconstitution. These recent approaches hold great promise for developing effective siRNA therapeutics [114, 115].

##### Cell-penetrating peptides (CPPs)

CPPs are a group of short peptides that can transport various biomolecules, including peptides, proteins, nucleic acids, and nanoparticles, across cellular membranes by penetrating the cell membrane [116]. The discovery of CPPs dates back to 1988 when arginine-rich peptides were identified in the HIV-1 Tat protein that facilitated its entry into cells and translocation to the nucleus [117]. Several CPPs have been identified, such as cationic peptides, which contain a high proportion of positively charged amino acids, and amphipathic peptides, which possess both hydrophobic and hydrophilic amino acids. Amphipathic peptides interact with the lipid bilayer to facilitate cellular uptake [118].

**Fig. 6:**
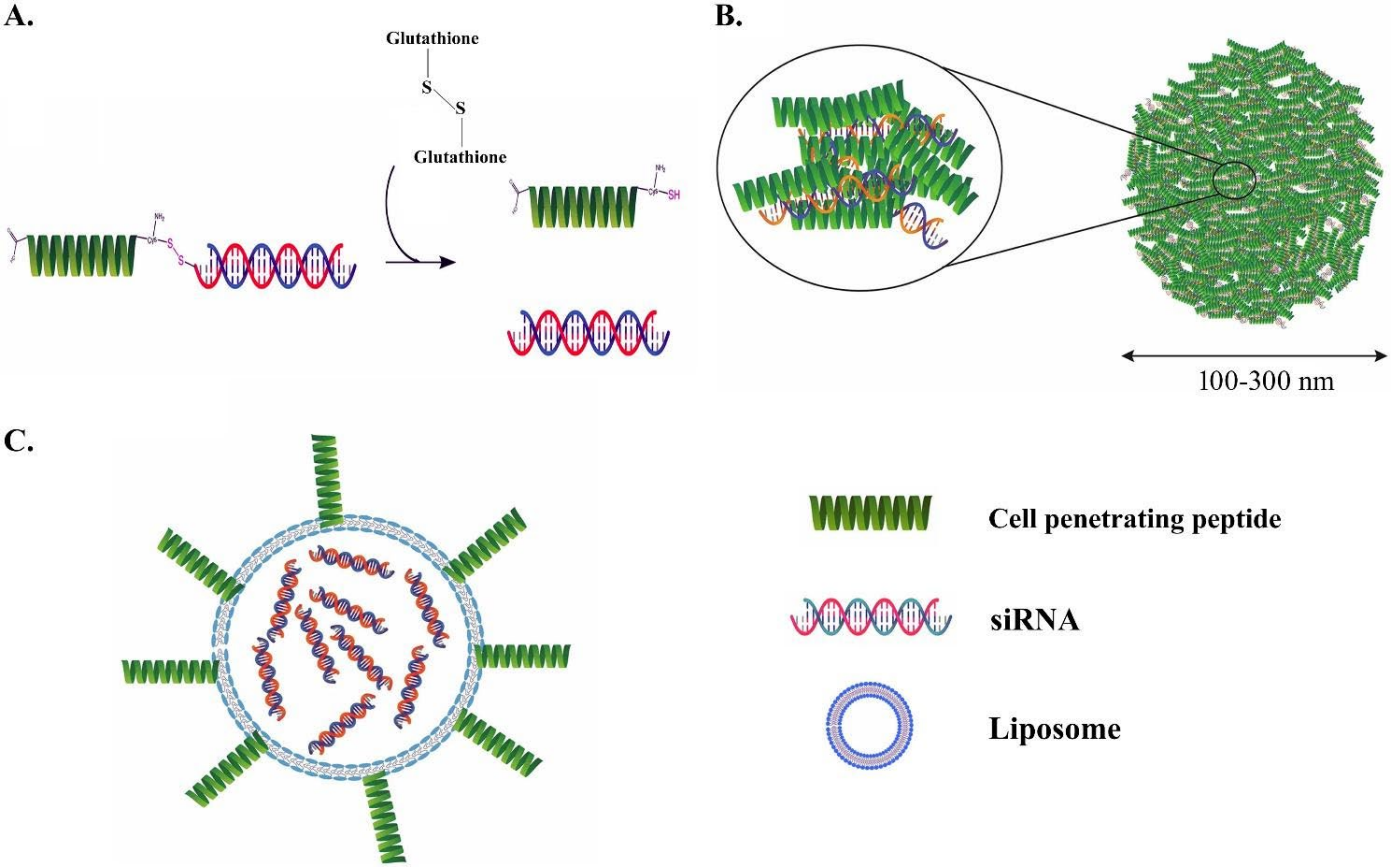
CPPs for siRNA delivery. **(A)** Covalently conjugated siRNA with CPP. **(B)** siRNA complexed with the CPP and **(C)** CPP-decorated nanoparticle. [116]

CPPs can transport siRNA across the cell membrane but lack specificity and may be taken up by non-target cells, reducing their effectiveness and causing toxicity [116, 119]. To increase specificity for target cells, CPPs can be conjugated with targeting ligands, such as antibodies or peptides (Fig. 6). This approach has been demonstrated in using an anti-EGFR antibody as a targeting ligand with CPP acting as an efficient facilitator of cellular uptake, resulting in specific delivery of siRNA to EGFR-positive cancer cells without impacting the viability or function of non-cancerous cells [120].

Another strategy is the use of CPPs modified with a pH-sensitive moiety that allows for selective release of the siRNA payload in the acidic tumor microenvironment, leading to enhanced gene silencing and inhibition of tumor growth [121]. Self-assembling peptides (SAPs) have also been utilized for siRNA encapsulation and protection to improve stability and pharmacokinetics [122, 123]. The use of multivalent SAPs, which contain multiple copies of the siRNA-binding motif, increases the affinity and specificity for siRNA, resulting in higher transfection efficiency and reduced off-target effects [124, 125]. SAPs can also be modified with targeting ligands that only bind to receptors located on the surface of target cells or tissues, improving siRNA transport to tumor cells and the effectiveness of the treatment [126].

Meade et al. developed a self-delivering siRNA platform composed of neutral phosphotriesters as internucleotide linkages that can be converted to negatively charged siRNA via cytoplasmic thioesterase. They conjugated hydrazine-containing peptide domains to siRNAs with aldehyde phosphotriester groups, which circumvented the loss of CPP activity observed in previous studies. In vitro experiments showed that these multi-Tat-siRNA conjugates effectively inhibited target protein expressions without the need for a transfection reagent. Increasing the number of multi-Tat peptide domains in the siRNA structure improved target protein silencing. However, this siRNA-peptide conjugate has not been applied in vivo [127].

##### Aptamers

Aptamer-mediated siRNA delivery is a promising approach for targeted delivery of siRNA molecules into cells. Aptamers are short, single-stranded nucleic acid molecules that possess high specificity and affinity for specific target molecules. By combining aptamers with siRNA, it is possible to achieve targeted delivery into cells, inducing gene silencing and therapeutic effects [128]. Compared to traditional delivery methods, aptamer-mediated siRNA delivery offers the advantages of specific cell and tissue targeting as well as sustained gene silencing, which may decrease the need for frequent dosing [129]. Aptamers have been designed to specifically target immune cells [130], cell surface receptors [129], and inflammatory markers [131] for siRNA delivery. The design of aptamers is critical for efficacy and specificity, and various approaches have been devised to improve aptamer-mediated siRNA delivery. These include multifunctional aptamer-siRNA conjugates [132] and stimuli-responsive aptamers [133]. However, developing aptamers with high specificity and affinity for their target molecules remains challenging.

Selecting aptamers that bind specifically to target cells or tissues is a crucial step in developing an aptamer-mediated siRNA delivery system. Recent developments in aptamer selection technologies, such as SELEX and high-throughput sequencing, have enabled the identification of aptamers with high affinity and specificity to various targets. Aptamer modification strategies, such as chemical modifications (e.g., LNAs or 2’-fluoropyrimidine modifications) and the addition of PEG moieties, have been explored to improve their binding affinity and specificity [132, 135]. Moreover, various conjugation strategies have been introduced, including thiol-modified aptamers and covalent linkages (e.g., click chemistry or amidation), to achieve stable and efficient siRNA conjugation to aptamers [136, 137].

Targeting is another aspect of aptamer-mediated siRNA delivery. The identification of targeting aptamers can be achieved through SELEX, which enables screening of oligonucleotide libraries for aptamers with high binding affinity to specific cells or tissues. In recent years, the field has made significant progress in enhancing the specificity and selectivity of aptamer-siRNA conjugates through novel targeting strategies [138]. One such approach involves the use of multiple aptamers to target distinct receptors on the same cell or tissue, thereby reducing the risk of off-target effects and improving specificity. An example is the aptamer-siRNA conjugate designed to target both EGFR and HER2 in breast cancer cells, which demonstrated enhanced selectivity and efficacy [138, 139]. Another strategy entails targeting tumor-associated antigens (TAAs), proteins that are overexpressed on the surface of cancer cells but absent on normal cells, utilizing aptamers that target TAAs can significantly enhance the specificity of the conjugate and reduce off-target effects. The aptamer-siRNA conjugate designed to target PSMA in prostate cancer cells has exhibited favorable outcomes in augmenting both selectivity and effectiveness [134].

##### Polymeric nanoparticles

Polymeric nano-carriers designed for targeted delivery of siRNA to specific cells offer a promising and innovative approach to overcome the challenges associated with traditional siRNA delivery methods, such as poor cellular uptake, low stability, and non-specific distribution throughout the body. The use of polymeric nanoparticles composed of biocompatible and biodegradable polymers, including PLGA, PEG, PLL, chitosan, and polyethyleneimine (PEI) presents a potential avenue for enhanced effectiveness and specificity of siRNA delivery, with minimized off-target effects, leading to safer and more effective treatments for various disorders. These nanoparticles can be engineered to encapsulate and protect siRNA from degradation, enhance cellular uptake, and allow controlled release of siRNA at the target site. Targeting ligands, such as peptides or antibodies, can also be incorporated into polymeric nanoparticles to enhance specificity and selectivity [106, 140–142].

In recent years, substantial progress has been made in the field of polymeric nanoparticles for siRNA delivery. New cationic polymers, such as PAMAM, PBAE, and PEI, have been developed and chemically modified to improve their efficiency and reduce their toxicity (Table 2). For instance, PAMAM has been modified with hydrophobic groups to improve its stability in serum, resulting in a potential increase in in vitro transfection efficiency of PAMAM-based siRNA delivery vectors [143]. Similarly, PBAE has been modified with oligopeptide linkages to decrease its toxicity while maintaining transfection efficiency. Modified PBAE polymers have shown efficient and low toxicity siRNA delivery to various cell types [144]. While PEI is widely used for siRNA delivery, owing to its unique attributes. PEI employs its greater charge density to establish strong electrostatic interaction with negatively charged siRNA molecules, providing efficient extracellular degradation protection. Of particular significance, PEI’s ability to regulate endosomal escape, an ability often ascribed to the well-documented “proton sponge effect”. Furthermore, PEI’s structural versatility, which includes the ability to fine-tune molecular weight, introduce branching modifications, and execute functionalization, makes it well-suited for the adaptation of delivery systems, addressing particular needs in the siRNA delivery domain. However, significant challenges emerge within this growing field, most notably the issue of cytotoxicity associated with high molecular weight PEI, which has prompted extensive research into alternative PEI variants engineered to limit toxicity, offering promising possibilities for safer siRNA delivery approaches [145]. Modified PEI polymers have been developed with improved efficacy and reduced toxicity, such as PEGylation of PEI to reduce toxicity while preserving its siRNA delivery capacity [146]. In addition, hybrid nanoparticles composed of both cationic polymers and lipids have been developed, improving stability, specificity, and efficacy of siRNA delivery [147].

Another significant advancement is the development of bioreducible polymers that can undergo degradation or breakdown in response to specific intracellular stimuli, such as reducing agents or reactive oxygen species. This leads to the release of siRNA into the cytoplasm of target cells, enhancing the specificity of siRNA delivery [148]. Bioreducible polymers have exhibited proficient siRNA delivery for anti-inflammatory therapy against myocardial ischemia-reperfusion injury, including disulfide-containing branched poly(β-amino ester) (SS-b-PAE) polymer for delivering siRNA to microvascular endothelial cells, which promoted therapeutic efficacy and reduced post-transfection toxicity [149]. Likewise, thiolated chitosan polymer was found to be biocompatible and effective in delivering siRNA to breast cancer cells in vitro and in vivo [150].

**Table 2:** Polymer NPs for the delivery of siRNA

Polymeric NPs	Study	Cell lines	Aid	Ref.
PEI-HYD	In vivo and in vitro	HUVECs	Targeted delivery	[151]
PLGA	In vitro	H1299	Improved encapsulation efficiency, high stability, highly resistant to nuclease degradation	[152]
PLGA-PLL-PEG	In vitro	HEK293T and A549	Site-specific delivery, improved endosomal escape and controlled release	[153]
Chitosan	In vivo and in vitro	H1299; NIH 3T3; mouse lung	Enhanced transfection efficiency, protection from nuclease degradation, low cytotoxicity	[154]
Chitosan-mPEG	In vivo	Mouse xenograft	Improved cell permeability and blood retention time	[155]

Moreover, core-shell hybrid nanoparticles have been synthesized using block copolymers, including PEG-b-PLA and PEG-b-PLGA, often combined with cationic lipids to encapsulate and release siRNA. For instance, Yang et al. employed PEG-b-PLA/BHEM-Chol nanoparticles to downregulate polo-like kinase 1 (Plk1) and suppress tumor growth in mice models [156, 157]. Moreover, PLGA-based block copolymer systems have been devised by Farokhzad and colleagues for systemic delivery of siRNA against a variety of targets, exhibiting effectiveness in a prostate cancer model [158].

## Conclusions and perspectives

Recent advancements in gene silencing have made it easier to develop plans for potential cures for various diseases. Among these advances, siRNA has emerged as a promising candidate with clinical trials yielding positive outcomes. Delivery of siRNA can be local, systemic, or both, but it is crucial to design siRNA so that it is not recognized as a foreign substance by the host system and can easily pass through extracellular and intracellular barriers that may hinder its effectiveness in vivo. The chemical modification of siRNA structure and conjugation with other molecules has made it possible to increase the half-life and potency of siRNA therapeutics while reducing off-target effects and enhancing stability. While delivery systems based on viral or non-viral vectors have shown acceptable efficiency with an overall satisfactory safety profile, the applications of siRNA therapeutics remain challenging and should not be underestimated. Nevertheless, the methods that have made substantial success in clinical applications are largely focused on delivering siRNA to well-vascularized tissues, utilizing endothelial fenestration that enables macromolecules to enter into target tissues. This approach has yielded remarkable results in diseases like cancer. Conversely, Delivering siRNA to less accessible tissues, such as solid tumors with dense stromal barriers, is still a formidable challenge. Consequently, the safe and efficient delivery of siRNA to a wide range of tissues, where its therapeutic potential exists, will almost certainly need the development of a diversified set of optimized systems. Each system should be tailored to address the specific challenges associated with delivering siRNA to a particular tissue, ensuring successful and efficient delivery. Furthermore, the use of membrane-disrupting molecules, which stay inactive throughout circulation but become active within the endosome, has shown efficacy. These well-established standards, however, are not immutable. These carriers appear to interact with the RNAi machinery via mysterious paths, which may include undiscovered intracellular trafficking pathways, undiscovered endosomal escape mechanisms, or even unusual non-endocytic cellular entry pathways. Research endeavors into diverse delivery platforms have the potential to elucidate biological processes that are now unclear and result in the development of new guiding principles as they proceed. Certainly, significant aspects of the delivery process are still ambiguous providing a favorable environment for innovative and cutting-edge strategies in the advancement of siRNA delivery materials.

## Data Availability

Not applicable.
